# Serum metabolic fingerprinting for diagnosis and therapeutic applications of ovarian endometriosis

**DOI:** 10.1016/j.isci.2026.114887

**Published:** 2026-02-02

**Authors:** Chencheng Dai, Yiwei Cao, Yiran Xu, Moyuan Li, Guangquan Liu, Sujuan Xu, Nuo Ye, Changxiang Shi, Tiantian Fan, Pengfei Xu, Xuemei Jia

**Affiliations:** 1 Nanjing Women and Children’s Healthcare Institute, Women’s Hospital of Nanjing Medical University (Nanjing Women and Children’s Healthcare Hospital), Nanjing 210004, P.R. China; 2 Department of Gynecology, Women’s Hospital of Nanjing Medical University (Nanjing Women and Children’s Healthcare Hospital), Nanjing 210004, P.R. China; 3 Nanjing Key Laboratory of Female Fertility Preservation and Restoration, Nanjing 210004, P.R. China; 4 The Affiliated Jiangning Hospital of Nanjing Medical University, Nanjing, P.R. China; 5 Department of Clinical Laboratory, Women’s Hospital of Nanjing Medical University (Nanjing Women and Children’s Healthcare Hospital), Nanjing 210004, P.R. China

**Keywords:** medicine, reproductive medicine, human metabolism

## Abstract

Ovarian endometriosis (OvE) is a gynecological disorder with endometrial tissue in the ovaries, for which effective non-invasive diagnosis and curative treatments are currently lacking. Serum samples were collected from both discovery and validation cohorts to examine the metabolomic signatures. Fifty-six differential metabolites between patients with OvE and healthy controls were identified using untargeted metabolomic profiling. Weighted gene co-expression network analysis was further conducted to validate the differential metabolites. Subsequently, twenty-one metabolites were selected for further validation using targeted metabolomic profiling. Five machine learning algorithms confirmed the efficacy and stability of these metabolites for diagnosing OvE. Least absolute shrinkage and selection operator -logit regression identified six serum metabolites and two clinicopathological features with high diagnostic accuracy. Three differential metabolites were found to exhibit therapeutic potential for OvE in an *in vivo* study. Diagnostic, predictive, and therapeutic potential of serum metabolomes for OvE are provided in this study.

## Highlights

• Serum metabolic fingerprinting in OvE is significantly altered
• A non-invasive diagnostic panel based on serum metabolic could distinguish OvE from HCs
• Selected differential metabolites show potential therapeutic prospects for OvE

## Graphical abstract

**Figure:**
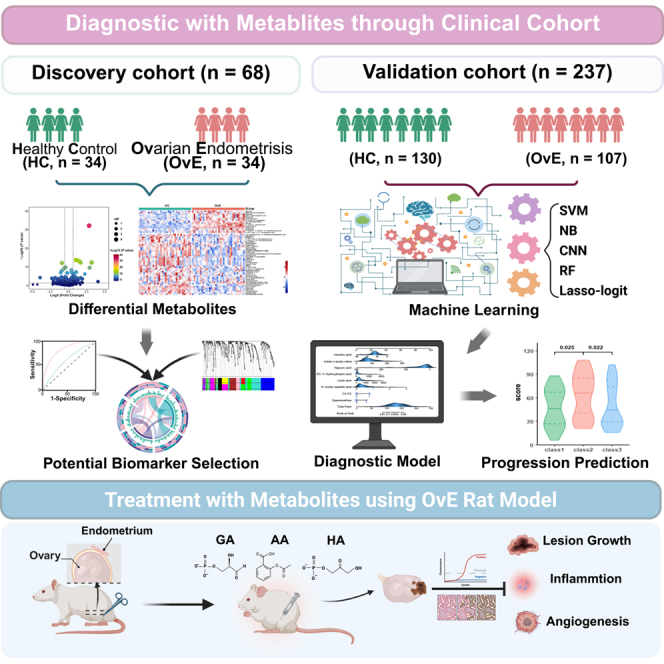

## Introduction

Endometriosis is defined as the presence of ectopic endometrial tissue outside the uterine cavity, such as in the ovaries, uterine ligaments, rectum, and bladder.^1^ Ovarian endometriosis (OvE), also known as chocolate cysts, is the most common form of endometriosis. It manifests primarily as dysmenorrhea, irregular menstruation, and pelvic pain and causes infertility in up to 50% of affected women.^2^ Additionally, women with endometriosis are prone to psychological and emotional disorders such as anxiety and depression.^3^ The incidence of OvE continues to increase, predominantly affecting younger women, and patients with OvE have a substantially higher risk of progression from a benign histological lesion to overtly invasive malignant tissue than those without OvE.^4^^,^^5^ Therefore, OvE poses a critical threat to the health of women.

An effective diagnosis is essential to enable intervention and prevent the development of OvE. However, OvE diagnosis remains challenging, usually resulting in a diagnostic delay of 5–12 years.^6^ This delay in OvE diagnosis may cause postponed treatment and poor prognosis and even increase the risk of recurrence and progression to endometriosis-associated ovarian cancer (EAOC).^4^^,^^7^ There are no reliable, non-invasive procedures to accurately diagnose OvE. Although serum carbohydrate antigen 125 (CA125) levels are correlated with OvE, the specificity and sensitivity remain unclear. Elevated CA125 levels are common in patients with severe endometriosis, strong pelvic inflammatory reactions, ovarian mass rupture, or adenomyosis.^8^ This highlights the urgency to develop a non-invasive diagnostic approach for OvE, particularly those based on serum biomarkers.

Metabolites represent endpoints of the transmission of biological information. Metabolites are located downstream of transcription and translation processes. Genome, transcriptome, and proteome modifications manifest as alterations in the metabolites.^9^ Many serum metabolites have shown potential for accurately diagnosing various diseases, such as cardiovascular disease, diabetes, inflammatory conditions, and cancer.^10^^,^^11^^,^^12^^,^^13^ In endometriosis, metabolites are derived from the plasma or serum,^14^^,^^15^^,^^16^ peritoneal cavity,^17^ follicular fluid,^18^ urine,^19^ the gut microbiome in particular,^20^^,^^21^ and focal ectopic tissue microbiomes.^22^ Therefore, metabolomics can potentially elucidate the pathogenesis of OvE and identify OvE biomarkers, even for EAOC.^23^ The aforementioned studies have been based on small cohorts, murine models, and different forms of endometriosis; whether such metabolites are promising in the diagnosis of endometriosis, particularly OvE, remains unclear.

Here, a high-resolution metabolic fingerprinting approach for OvE diagnosis is reported. A comprehensive analysis of the serum metabolites in patients with OvE and healthy control (HC) females was conducted using untargeted metabolomics and weighted gene correlation network analysis (WGCNA) in a discovery cohort. Targeted metabolic quantification analysis was used to validate metabolite levels in the validation cohort with more OvE and HC individuals. Five machine learning algorithms were then used to assess the diagnostic performance of these metabolites in identifying OvE. A diagnostic model for OvE based on the selected metabolites and clinical features was established. Metabolites were also used to predict OvE severity. Three decreased metabolites (gallic acid [GA], ascorbic acid [AA], and hippuric acid [HA]) were selected that offered therapeutic potential for alleviating proliferation, inflammation, and angiogenesis in a rat OvE model. The findings suggest that serum metabolite profiling is effective for the diagnosis, prediction, and development of treatment strategies for OvE.

## Results

### Metabolic profiling reveals altered metabolites in OvE

**Figure 1:**
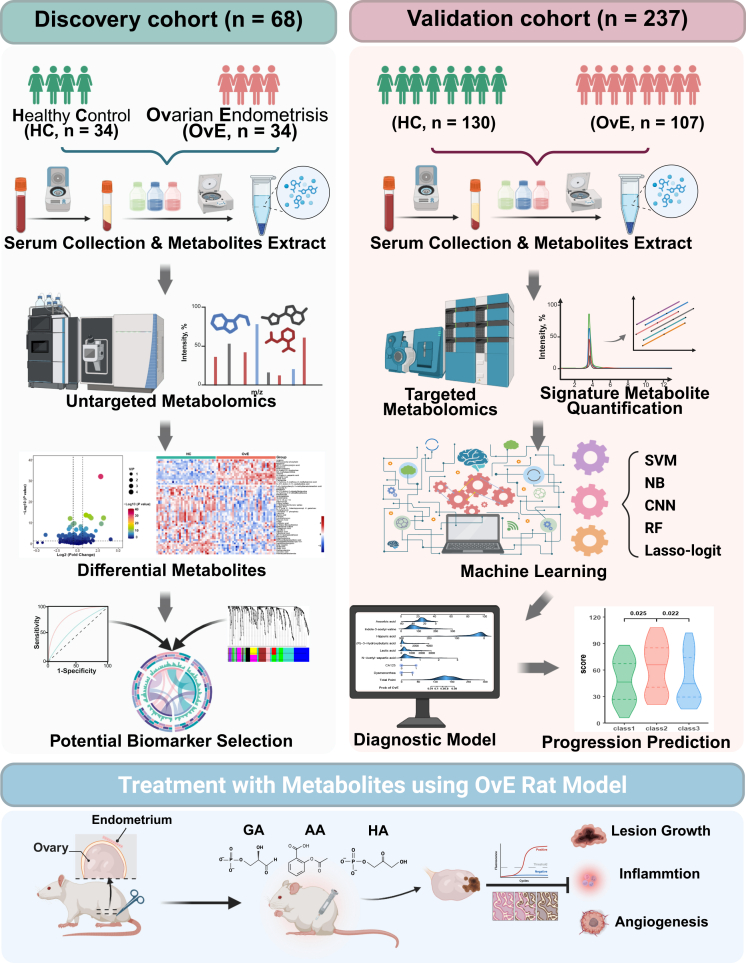
Schematic workflow diagram of our study Serum from individuals with OvE or HC were collected and subjected to untargeted metabolomics. Then WGCNA was used to identify the key metabolites associated with OvE. Subsequently, targeted metabolomics was utilized to identify differentially expressed metabolites based on the intersection of WGCNA and untargeted metabolomics. Five machine learning algorithms were applied to evaluate the efficacy of those metabolites. Ultimately, a panel metabolites diagnostic model was conducted for OvE. Additionally, three selected metabolites were found to exhibit therapeutic potential for OvE *in vivo* study. This figure was created in BioRender (https://BioRender.com/d76n145).

To identify specific metabolites in women with OvE, serum samples from the discovery and validation cohorts were collected to perform a metabolic profile workflow (Figure 1). Detailed demographic and clinical features of women with and without OvE are provided in Table 1.

**Table 1:** The clinical pathological of discovery and validation cohorts

Clinicopathological Features	Discovery cohort			Validation cohort		
	HC (*N* = 34)	OvE (*N* = 34)	*p*	HC (*N* = 130)	OvE (*N* = 107)	*p*^*a*^
Age (years)	31.76 ± 7.78	31.97 ± 7.26	0.91	34.63 ± 8.15	33.11 ± 6.80	0.127
BMI	21.36 ± 1.62	20.78 ± 2.24	0.223	22.20 ± 2.97	21.61 ± 3.04	0.130
Dysmenorrhea						
No	**32 (94.12%)**	**18 (52.94%)**	**<0.001**	**120 (92.30%)**	**35 (32.71%)**	**<0.001**
Yes	**2 (5.88%)**	**16 (47.06%)**		**10 (7.70%)**	**72 (67.29%)**	
WBC (10^9^/L)	6.14 ± 1.81	5.72 ± 1.32	0.274	**6.49** ± **1.54**	**5.93** ± **1.84**	**0.014**
NEUT (10^9^/L)	3.61 ± 1.60	3.74 ± 1.30	0.715	3.84 ± 1.27	3.87 ± 1.80	0.871
MONO (10^9^/L)	0.314 ± 0.12	0.30 ± 0.10	0.401	**0.34** ± **0.11**	**0.31** ± **0.11**	**0.008**
CHOL (mmol/L)	**4.75** ± **0.83**	**4.36** ± **0.57**	**0.028**	**4.92** ± **1.00**	**4.67** ± **0.80**	**0.037**
TG (mmol/L)	**1.09** ± **0.57**	**0.76** ± **0.36**	**0.006**	**1.17** ± **0.70**	**0.85** ± **0.45**	**<0.001**
ALT (U/L)	17.13 ± 8.7	16.46 ± 10.71	0.777	18.54 ± 16.18	16.62 ± 9.62	0.281
AST (U/L)	19.01 ± 3.54	18.34 ± 5.87	0.572	19.75 ± 5.73	18.61 ± 4.95	0.106
GGT (U/L)	15.53 ± 3.82	17.69 ± 12.08	0.324	20.19 ± 13.67	17.86 ± 10.29	0.146
HDL_C (mmol/L)	1.71 ± 0.28	1.68 ± 0.40	0.741	1.68 ± 0.40	1.75 ± 0.50	0.25
AFP (ng/mL)	**1.97** ± **1.91**	**2.76** ± **1.13**	**0.049**	7.22 ± 44.46	2.69 ± 1.14	0.294
CA199 (U/mL)	**9.37** ± **5.85**	**33.32** ± **39.48**	**0.002**	**10.77** ± **10.76**	**35.37** ± **37.92**	**<0.001**
CEA (ng/mL)	0.90 ± 0.82	1.16 ± 0.47	0.134	1.09 ± 0.94	1.13 ± 0.59	0.649
CA125 (U/mL)						
Normal (<35)	**34 (100%)**	**9 (26.47%)**	**<0.001**	**125 (96.00%)**	**37 (34.58%)**	**<0.001**
High (>35)	**0 (0%)**	**25 (73.53%)**		**5 (4.00%)**	**70 (65.42%)**	
ASRM stages						
II (scores)	N/A	**0**	N/A	N/A	**1 (6)**	N/A
III (scores)	N/A	**13 (28.88** ± **5.89)**		N/A	**51 (26.08** ± **9.80)**	
IV (scores)	N/A	**21 (81.17** ± **21.03)**		N/A	**55 (85.19** ± **16.77)**	

For continuous variables, we presented the data as mean and standard deviations (SDs). Categorical variables were presented as counts (proportions). BMI, body mass index; WBC, white blood cell; NEUT, neutrophils; MONO, monocytes; CHOL, cholesterol; TG, triglycerides; ALT, alanine aminotransferase; AST, aspartate aminotransferase; GGT, γ-glutamyl transferase; HDL_C, high-density lipoprotein cholesterol; AFP, α-Fetoprotein; CA199, carbohydrate antigen 19-9; CEA, carcinoembryonic antigen; CA125, cancer antigen 125; ASRM, American Society for Reproductive Medicine; N/A, no appliance; ^a^*p* were determined by the chi-squared test or Fisher’s exact test for categorical variables and paired *t* tests or Wilcoxon’s signed-rank test for continuous variables. The bold values indicated these data are statistically different.

**Figure 2:**
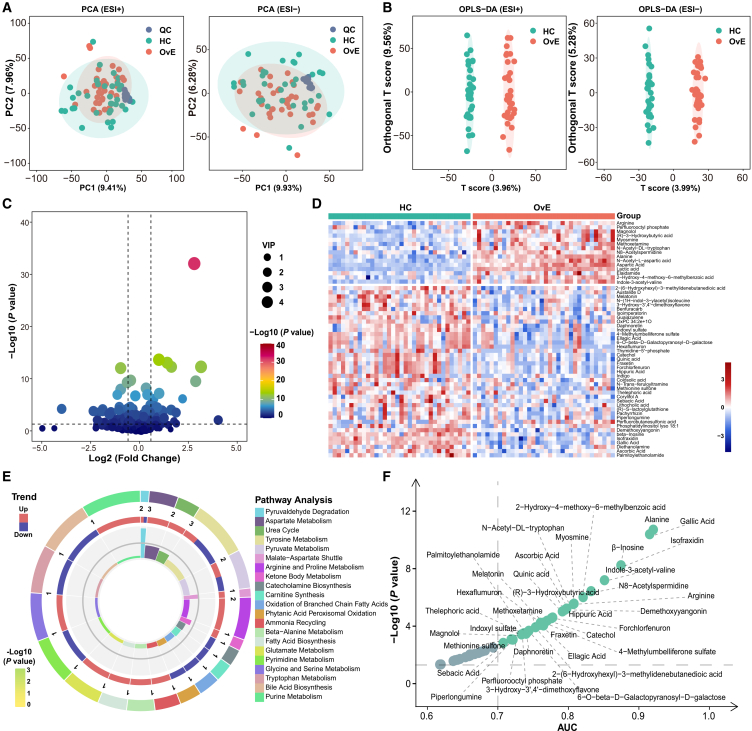
Untargeted metabolomics of serum and identification of alteration of metabolites in OvE (A) PCA plots of samples from two groups in negative and positive ion models. QC, quality control. (B) OPLS-DA plots of samples from two groups in negative and positive ion models. (C) Volcano plot of identified metabolites in the datasets. The *x* axis indicates log2 (fold change) while the *y* axis indicates -log10 (*p* value). (D) Heatmap of the relative abundance of the metabolites differentially expressed between OvE and HC groups. (E) Metabolic pathway analysis of differential expressed metabolites by MSEA. (F) ROC curve performance of differential metabolites (AUC >0.7) determined from OvE patients. The *x* axis indicates AUC while the y axis indicates -log10 (*p* value).

Untargeted metabolomics was used to examine metabolomic profiles in the discovery cohort. In the original data, the two ion modes yielded 11,394 metabolite features, of which 5,153 and 6,241 were in the positive and negative ion modes, respectively. Principal-component analysis (PCA) indicated that in both positive and negative ion mode, the quality control samples were highly aggregated, demonstrating that the experiment was stable and the data were reliable (Figure 2A). Orthogonal partial least squares-discriminant analysis (OPLS-DA) score plots indicated substantial differences in serum metabolites between the two groups in the positive and negative ion modes (Figure 2B). These data suggest that patients with OvE experience more metabolic disorders than HCs. A total of 612 metabolites were eventually included in the analysis. For further analysis with fold change (FC) > 2, *p* < 0.05, and variable importance of projection (VIP) > 1.5, 56 metabolites that were markedly different between the two groups were identified, of which 16 were upregulated, and 40 were downregulated in the OvE group (Figures 2C and 2D). Metabolite set enrichment analysis (MSEA) and Kyoto Encyclopedia of Genes and Genomes (KEGG) pathway analysis indicated the involvement of these differential metabolites in pathways, including pyruvate, β-alanine, arginine, proline, pyrimidine, and tryptophan metabolism (Figures 2E and S1). To identify specific metabolites for diagnosing OvE, a receiver operating curve (ROC) curve analysis of these differential metabolites was performed. There were 32 metabolites exhibiting the area under curve (AUC) > 0.7 (Figure 2F).

### WGCNA reveals potential metabolic module in OvE

**Figure 3:**
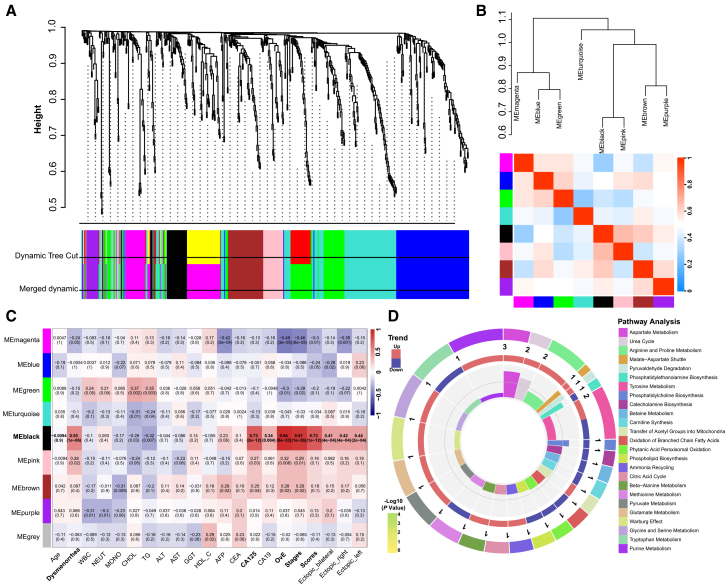
WGCNA analysis and identification of the key metabolites’ module of OvE (A) Clustering dendrogram of the average network adjacency to identify metabolite co-expression modules. (B) Dependencies between modules. (C) Heatmap of module-trait relationships, analyzing correlations between metabolites and clinicopathological features. (D) Metabolic pathway analysis of the metabolites of black module by MSEA.

WGCNA was conducted to further identify the metabolites that were more closely associated with OvE. First, the relationship between metabolites and clinical features was analyzed and the metabolites were categorized into nine modules based on distinct expression patterns (Figures 3A and 3B). The metabolites within the black module exhibited strong correlations with OvE characteristics, including dysmenorrhea (R = 0.55, *p* < 0.001), high CA125 levels (R = 0.73, *p* < 0.001), ovarian cysts identified using ultrasound (R = 0.94, *p* < 0.001), and the American Society for Reproductive Medicine (ASRM) laparoscopy stage (R = 0.91, *p* < 0.001) and scores (R = 0.73, *p* < 0.001) (Figure 3C). Further, the MSEA and KEGG pathway analyses indicated that these differential metabolites within the black module were primarily associated with the citric acid cycle, and β-alanine, pyrimidine, glutamate, arginine, and proline metabolisms (Figures 3D and S2). The WGCNA indicated that the metabolites in the black module might be associated with OvE.

### Targeted metabolomics validates selected metabolites in OvE

**Figure 4:**
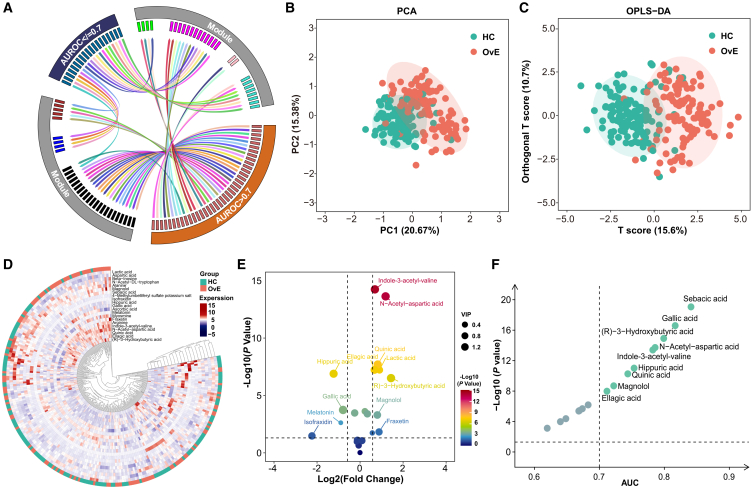
Targeted metabolomics of selected metabolites (A) Contact tracing circus plot highlighting the 21 intersected metabolites between the metabolites in black module and those of AUC >0.7 in untargeted metabolomics. (B–C) PCA and OPLS-DA plots of samples from two groups. (D) Heatmap of the relative abundance of the metabolites differentially expressed between OvE and HC groups. (E) Volcano plot of identified metabolites in the datasets. The *x* axis indicates log2 (fold change) while the *y* axis indicates -log10 (*p* value). (F) ROC curve performance of differential metabolites (AUC >0.7) determined from OvE patients.

To identify the most important metabolites in OvE, an intersection analysis between the untargeted metabolites with AUC >0.7 and those in the black module of WGCNA was conducted (Figure 4A). Twenty-one metabolites were identified for further analysis in 107 patients with OvE and 130 HCs (Figure S3). PCA and OPLS-DA showed that all metabolites could be differentiated (Figures 4B and 4C). The results indicated 16 metabolites that were differently expressed, of which 12 were increased and 4 were decreased in patients with OvE (Figures 4D, 4E, and S4). Of these, nine had an AUC of >0.7 (Figure 4F). Metabolomics identified serum metabolites that were distinct in women with OvE.

### Diagnostic potency of OvE-associated metabolites evaluated via machine learning algorithms

**Figure 5:**
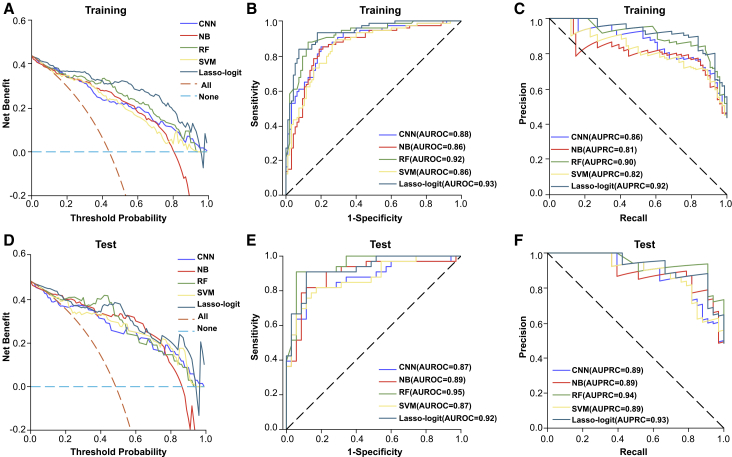
Machine learning algorithms predicted the diagnostic potency of the selected metabolites (A) Decision curve analysis from training set for selected metabolites by CNN, NB, RF, SVM, and LASSO-logit analysis. The *x* axis represented the range of threshold probabilities and the *y* axis represented net benefit. The closer the curve as a whole was to the upper right corner, the better the predictive performance of the model. (B) ROC curve from training set performance of the selected metabolites by CNN (cutoff = 0.263, sensitivity = 85.1%, and specificity = 80.0%), NB (cutoff = 0.325, sensitivity = 83.8%, and specificity = 81.1%), RF (cutoff = 0.410, sensitivity = 87.8%, and specificity = 87.4%), SVM (cutoff = 0.273, sensitivity = 89.2%, and specificity = 71.6%), LASSO-logit (cutoff = 0.521, sensitivity = 83.8%, and specificity = 92.6%). (C) PRC curve from training set performance of the selected metabolites by CNN (cutoff = 0.263; recall = 85.1% [consistent with sensitivity], and precision = 76.8%), NB (cutoff = 0.325; recall = 83.8%, and precision = 77.5%), RF (cutoff = 0.410; recall = 87.8%, and precision = 84.4%), SVM (cutoff = 0.273; recall = 89.2%, and precision = 71.0%) and LASSO-logit (cutoff = 0.521; recall = 83.8%, and precision = 89.9%). (D) Decision curve analysis from test set for selected metabolites by CNN, NB, RF, SVM, and LASSO-logit analysis. (E) ROC from test set performance of the selected metabolites by CNN (cutoff = 0.298; sensitivity = 81.8%, and specificity = 88.6%), NB (cutoff = 0.514; sensitivity = 81.8%, and specificity = 88.6%), RF (cutoff = 0.431; sensitivity = 90.9%, and specificity = 94.3%), SVM (cutoff = 0.386; sensitivity = 81.8%, and specificity = 82.9%), and LASSO-logit (cutoff = 0.495; sensitivity = 90.9%, and specificity = 88.6%); (F) PRC from test set performance of the selected metabolites by CNN (cutoff = 0.298; recall = 81.8%, and precision = 87.1%), NB (cutoff = 0.513; recall = 81.8%, and precision = 87.1%), RF (cutoff = 0.431; recall = 90.9%, and precision = 93.8%), SVM (cutoff = 0.386; recall = 81.8%, precision = 81.8%) and LASSO-logit (cutoff = 0.495; recall = 90.9%, and precision = 88.2%).

Subsequently, the potential diagnostic values of these 21 metabolites for OvE were determined. Five machine learning algorithms, convolutional neural network (CNN), naive bayes (NB) algorithm, random forest (RF), support vector machine (SVM), and least absolute shrinkage and selection operator (LASSO)-logit, were used. The decision curve analysis indicated that the algorithm achieved a substantial net benefit in the training and test sets (Figures 5A and 5D). ROC analysis indicated that the diagnostic model exhibited an AUC of ROC (AUROC) of 0.86–0.93 in the training set and 0.87–0.95 in the test set (Figures 5B and 5C). The precision-recall curve (PRC) analysis demonstrated that the model’s AUC of PRC (AUPRC) varied from 0.81 to 0.92 in the training set and from 0.89 to 0.94 in the test set (Figures 5E and 5F). These findings suggest that these 21 metabolites can be used to reliably diagnose OvE.

### Metabolite-based OvE diagnostic model construction and validation

**Figure 6:**
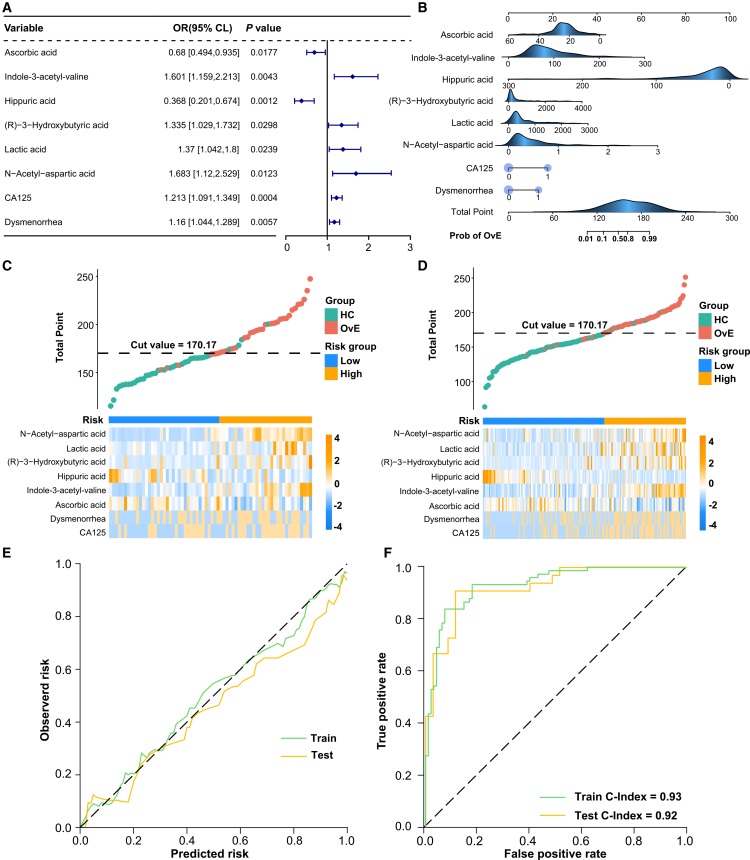
The diagnostic model for OvE construction and validation (A) Forest plot of the multivariate Lasso logistic regression analyses based on the validation cohort. (B) Nomogram for the diagnosis of OvE. Blue density plots represented the distribution of continuous variables. (C–D) Distribution of risk score and the expression of the selected metabolites involved in the formation of the risk score. A threshold point value was defined as 170.17, and all participants were divided into low-risk (<cutoff) and high-risk (>cutoff) score groups from training and test set. (E–F) Consistency evaluation indicated a C-index of 0.93 for the training set and 0.92 for the test set.

LASSO-logit analyses were conducted on 21 metabolites and 13 clinical indicators (Figure S5) to produce a panel of six metabolites (including N-acetyl-aspartic acid, lactic acid, (R)-3-hydroxybutyric acid, HA, indole-3-acetyl-valine, and AA), and two clinical indicators (CA125 and dysmenorrhea), which showed good performance in differentiating patients with OvE from HCs. The six metabolites and two clinical features were subsequently subjected to Cox proportional hazards regression analysis. The forest plot of multivariate LASSO-logit produced a strong correlation between these eight indicators and OvE (Figure 6A). Therefore, these eight indicators were integrated into the diagnostic model. The nomogram showed a specific concentration range and corresponding score ranges for each metabolite in the OvE diagnostic model (Figure 6B).

Using this OvE diagnostic model, the risk score for each patient in the training and test sets was calculated, assigning patients to high- or low-risk groups based on a median risk score of 170.17 (Figures 6C and 6D). Using this diagnostic model to predict OvE risk, confusion matrices were obtained. In the training set, the true-positive rate for the diagnosis of OvE was 61 out of 74 (82.43%), and the false-positive rate of healthy women misdiagnosed with OvE was 7 out of 95 (7.37%). In the test set, the true-positive rate for the diagnosis of OvE was 3 out of 33 (90.91%), and the false-positive rate of healthy women misdiagnosed with OvE was 1 out of 35 (2.86%) (Figure S6). The calibration curve showed that nomogram predictions closely matched the observed results (Figure 6E). Consistency evaluation indicated a concordance index (C-index) of 0.93 and 0.92 for the training and test sets, respectively (Figure 6F).

The diagnostic efficiency of the six metabolites and two clinical features was determined. Decision curve analysis demonstrated that the model based on the eight indicators provided a higher net benefit than two clinical features alone in both the training and test sets (Figures S7A and S7D). The ROC analysis indicated that the diagnostic model in the training set had an AUROC of 0.92, 0.67, and 0.93 for the six metabolites, two clinical features, and the eight indicators, respectively; in the test set, the AUROCs were 0.91, 0.67, and 0.92, respectively (Figures S7B and S7E). The PRC analysis indicated AUPRC values of 0.64, 0.90, and 0.92 for the training set and 0.70, 0.87, and 0.88 for the test sets, respectively (Figures S7C and S7F). These findings indicate that serum metabolites are good diagnostic indicators of OvE.

### OvE severity prediction using metabolites

Whether targeted metabolite fingerprinting could distinguish the OvE subgroups was determined. Patients with OvE were divided into three subgroups according to uniform manifold approximation and projection (UMAP) (Figure S8A). The ASRM stages and scores of class II were higher than those of classes I and III (Figure S8B). The ASRM scoring primarily included the location, number, size, and adhesion degree of the ectopic endometrial lesions. Hence, the number and size of ectopic endometrial lesions in the three subgroups were analyzed. The number and diameter of the lesion of class II ectopic endometrial lesions were similar to those of classes I and III (Figures S8C and S8D). Thus, these results suggest that metabolite fingerprints can also be used to predict the progression of OvE, especially, the degree of adhesion.

### OvE therapeutic potential exploration using metabolites

**Figure 7:**
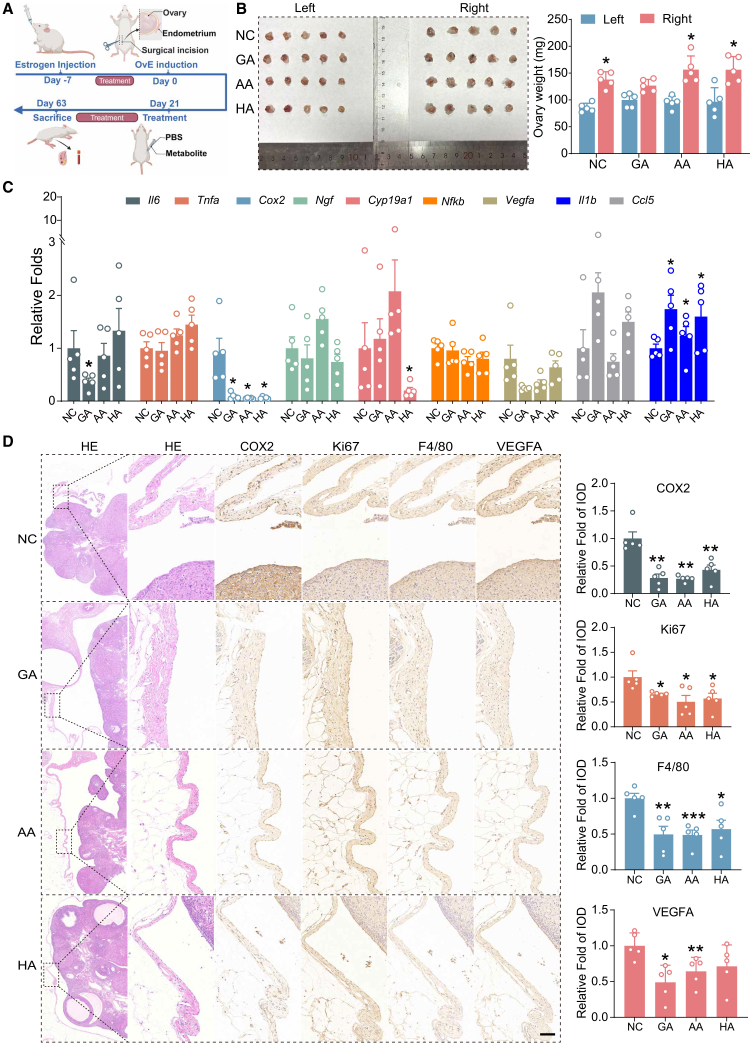
Metabolites suppress the progression of OvE *in vivo* (A) Schematic representation of experimental timeline and procedures in rat OvE model. This figure was created in BioRender (https://BioRender.com/rlasz0x). (B) The normal ovaries and OvE lesions from NC, GA, AA, and HA groups. The masses of lesions from the indicated treatment groups are shown. Data are presented as mean ± SEM (*n* = 5). Asterisks indicate significance between groups: ∗*p* < 0.05. (C) Expression of endometriosis-related cytokines in OvE tissues were analyzed by qPCR. The mRNA levels are expressed relative to transcript level in normal ovary tissue, set at 1.0 (*n* = 5 per group). Asterisks indicate significance between groups: ∗*p* < 0.05; (D) Representative H&E-stained sections of lesions and sections stained with anti-COX2 (inflammation), anti-Ki67 (proliferation), anti-F4/80 (macrophages and inflammation), and anti-VEGFA (angiogenesis). Data are presented as mean ± SEM (*n* = 5). Asterisks indicate significance between groups: ∗*p* < 0.05, ∗∗*p* < 0.01, ∗∗∗*p* < 0.001. Scale bars, 100 mm.

To find potential metabolites that could be used as a treatment or an adjuvant therapy for OvE, three metabolites—AA, GA, and HA—were selected based on their significant downregulation in patients with OvE and their potential anti-inflammatory effects. The rat model of OvE was divided into four groups and rats were administered the differential metabolites (AA, GA, HA, and NC) intraperitoneally (Figure 7A). After 6 weeks of administration, the rats were anesthetized using CO_2_ inhalation (Figure S9A). There was no marked difference in body weight or serum estradiol between the four groups (Figures S9B and S9C). For each group, the weights of the right ovaries with OvE were substantially greater than those of the normal left ovaries (Figure 7B).

To detect the expression of OvE-related genes, such as the estrogen-related gene, *Cyp19a1*, and inflammation-related genes (*Il6*, *Tnf*a, *Cox2*, *Ngf*, *Nfkb1*, *Vegfa*, *Il-1b*, and *Ccl5)*, RNA were extracted from the OvE tissues. The quantitative polymerase chain reaction (qPCR) results indicated that *Il6* was reduced in the GA group, *Cyp19a1* was reduced in the HA group, *Cox2* was reduced in all three metabolites groups, whereas the anti-inflammatory gene, *Il-1b*, was substantially increased in all the three groups in comparison to the control group (Figure 7C). The selected metabolites, AA, GA, and HA, all exhibited different inhibitory effects on the inflammatory environment of OvE.

Pathological examination indicated the presence of ectopic endometrium on the right ovary, which indicated the OvE model in rats was successfully established. Immunohistochemical (IHC) staining detected the expression of proliferation (Ki67), inflammatory (Cyclooxygenase-2, COX2), macrophage (F4/80), and angiogenesis markers (Vascular Endothelial Growth Factor A, VEGFA), which were all reduced in the OvE lesions of three metabolites groups compared to the controls (Figure 7D). It was therefore concluded that these three metabolites (AA, GA, and HA) could substantially alleviate the proliferation, inflammation, and angiogenesis of OvE in rats.

## Discussion

OvE, a primary form of endometriosis, adversely affects the quality of life of women. A delayed OvE diagnosis substantially affects the prognosis and increases the risk of recurrence and occurrence of EAOC.^4^ Metabolites are substantially altered in women with endometriosis, including OvE. Moreover, metabolites from the serum or gut microbiome are promising non-invasive diagnostic tools for endometriosis. In the current study, an untargeted metabolomic analysis of the discovery cohort with 68 individuals was conducted. A targeted metabolic quantification analysis of a validation cohort with 237 participants was conducted. A diagnostic scoring model was developed based on six metabolites (including N-acetyl-aspartic acid, lactic acid, (R)-3-hydroxybutyric acid, HA, indole-3-acetyl-valine, and ascorbic acid), as well as two clinical features (CA125 and dysmenorrhea), which exhibited a strong diagnostic capability for OvE. The combination of these metabolites with clinical features greatly improved the diagnostic accuracy of OvE compared to that of the clinical features alone. These targeted metabolites can potentially predict OvE severity, particularly the degree of adhesion. Finally, three decreased metabolites (GA, AA, and HA) were selected, which offered therapeutic potential for alleviating proliferation, inflammation, and angiogenesis in a rat OvE model.

Differences in the gut microbiota and microbiota-derived metabolites between individuals with or without endometriosis have been reported.^20^^,^^21^ Furthermore, distinct stool metabolites may influence the development of endometriosis and are effective for non-invasive diagnosis.^20^^,^^24^ In the current study, several distinct metabolites were derived from the gut microbiota, including quinic acid, GA, and some amino acids. However, quinic acid showed decreased concentrations in OvE patients in the current study, whereas Chadchan et al. reported increased concentrations in a murine endometriosis model.^21^ The different results for quinic acid are possibly due to differences in the samples, species, and endometriosis types. Human serum was used to determine metabolites through untargeted metabolomics and further validated using targeted metabolomic measurements in this study, whereas Chadchan et al. assessed metabolites using the stool of antibiotic-induced microbiota-depleted mice. It is possible that the metabolites in the current study originated from host-microbe co-metabolism. Only OvE was investigated, which is one form of endometriosis characterized by the ectopic presence of endometrial tissue in the ovaries. However, Chadchan et al. used an endometriosis model where uterine tissue from estrus-stage mice was autologously transplanted into the peritoneal wall. It is possible that different types of endometriosis may contain different metabolites.

HA levels were significantly decreased in the OvE group, consistent with a previous study.^20^ As a host-microbiota co-metabolite, HA is synthesized in the liver and kidneys through the conjugation of benzoic acid with glycine and is subsequently excreted in urine.^25^^,^^26^ As such, circulating HA levels reflects the combined impact of gut microbiome composition, host metabolic capacity, and renal excretion. Accumulating evidence from physiological and pathological states indicates that HA’s effects are highly context dependent. In healthy aging, HA levels rise, whereas impaired HA excretion has been noted in aging-related conditions—highlighting that HA dynamics are sensitive to systemic metabolic status.^27^ Beyond acting as a metabolic marker, HA also directly regulates tissue homeostasis by inhibiting osteoclastogenesis and bone resorption.^28^ Notably, HA has been shown to exert opposing effects under different pathological circumstances. In diseases defined by prominent oxidative stress and innate immune activation, elevated HA is linked to oxidative damage and inflammatory signaling—observed in vitiligo, chronic kidney disease, and cocaine-induced inflammatory responses.^29^^,^^30^^,^^31^ In contrast, in chronic inflammatory diseases driven by metabolic-immune dysregulation, HA appears to offer protective effects. Reduced HA levels are consistently reported in inflammatory bowel disease (IBD), and HA supplementation eases colonic inflammation and supports intestinal metabolic homeostasis.^32^^,^^33^ OvE shares key pathophysiological features with IBD, including chronic inflammation and growing links to gut microbiome alterations. This similarity is further supported by metabolomic studies showing a strong correlation between fecal metabolite profiles in endometriosis and IBD.^20^ Within this framework, reduced HA in OvE likely reflect the loss of a microbiota-associated anti-inflammatory metabolite rather than pathological accumulation. This interpretation is supported by evidence that HA supplementation improves ovarian function in polycystic ovary syndrome models and is positively associated with cardiometabolic health.^34^^,^^35^ Additionally, HA has been used as a metaphylactic agent in calcium oxalate lithiasis, highlighting its diverse biological properties.^36^ Taken together, these findings suggest that the varied effects of HA across diseases are primarily shaped by disease context, inflammatory load, microbiome composition, and HA metabolism and clearance. In OvE, decreased HA levels may therefore represent a maladaptive metabolic signature linked to chronic inflammation and disrupted host-microbe interactions.

Indole-3-acetyl-valine belongs to the class of organic compounds known as N-acyl-alpha amino acids. Although it is reported as a native auxin conjugate of liverwort,^37^ it is also found in the Human Metabolome database (https://hmdb.ca/). Many plant-derived metabolites in human serum, including GA^38^ and quinic acid,^21^ have been reported to potentially originate from the gut microbiota. What is more, previous studies had reported that there are significant differences in the gut microbiota of patients with endometriosis.^20^^,^^21^ Therefore, we hypothesize that indole-3-acetyl-valine may be derived from the gut microbiota. Regarding the function of indole-3-acetyl-valine in OvE, Marabotti et al. reported that indole-3-acetyl-valine is the allosteric effectors of tryptophan synthase, which catalyze the formation of L-tryptophan.^39^ L-tryptophan serves as a precursor for the synthesis of 5-hydroxytryptamine and melatonin. L-tryptophan has been implicated in schizophrenia,^40^ Alzheimer’s disease,^41^ autism,^42^ and epilepsy.^43^ Therefore, indole-3-acetyl-valine may promote neurological growth. Nerve fibers,^44^^,^^45^ brain-derived neurotrophic factor, and neurotrophin-3^46^ are increased in endometriotic lesions, and may contribute to the etiology of neuroangiogenesis and endometriosis-associated pelvic pain.^47^ In addition to indole-3-acetyl-valine, (R)-3-hydroxybutyric acid, a metabolite closely related to nerves,^40^^,^^41^^,^^48^ was markedly upregulated in the OvE group of this study. It has been found that (R)-3-hydroxybutyric acid is significantly increased in patients with Budd-Chiari syndrome and adenomatous polyposis coli (APC) gene mutation of patients with intestinal adenomatous polyps.^49^^,^^50^ The gut microbiota of these patients is severely disrupted. Therefore, (R)-3-hydroxybutyric acid may also be derived from the gut microbiota. (R)-3-hydroxybutyric acid is involved in the synthesis and degradation of ketone bodies and can be used as an energy source in the brain during hypoglycemia. Therefore, nerves and their associated metabolites may be associated with OvE, and the high expression of nerve-associated metabolites may be a good marker for OvE diagnosis.

In the current study, lactic acid levels were upregulated in the OvE group. This finding is consistent with that of two previous studies, which indicated that lactic acid content was elevated in the serum of women with endometriosis^51^ and in the stool of endometriotic mice.^21^ Lactic acid, an end product of the glycolytic pathway, elevates the expression of lactate dehydrogenase, lipid peroxidase, and other glycolysis-related proteins in endometriosis^52^ and substantially influences the crosstalk between endothelial cells and the immune microenvironments in endometriosis.^53^ Additionally, lactic acid has been implicated as a crucial factor in lactation modification^54^^,^^55^^,^^56^ and pH-dependent suppression of immune cell function.^57^ Given its molecular roles in other diseases, the elevated concentrations of lactic acid in the serum of patients with OvE may contribute to its increased role in OvE. This requires further investigation.

Oxidative stress is a well-established critical factor in the pathogenesis of OvE,^58^ and OvE severity is usually positively correlated with oxidative stress.^59^^,^^60^ Antioxidant therapies such as vitamin supplementation have demonstrated efficacy in downregulating or inhibiting certain inflammatory cytokines, which show promise in relieving dysmenorrhea and pelvic pain in patients with OvE.^58^^,^^61^ Notably, we observed decreased contents of vitamin C (AA) in the OvE cohort. Intravenous vitamin C treatment in a rat model of endometriosis was reported to suppress the prevention of endometriotic implant induction and regression of endometriotic implant volumes.^62^ Consequently, assessing vitamin C levels could have implications for OvE diagnosis and treatment.

OvE progression and metabolic disturbances are closely related and mutually causal. Women with a low body mass index (BMI) are more susceptible to developing OvE.^63^ In this study, women with OvE tended to have a lower BMI than HCs. Endometriosis affects the metabolism in the liver and fat tissues, causes systemic inflammation, and alters the expression of the brain-expressed genes that cause pain sensitivity and mood disorders.^64^ One study focused on young patients with endometriosis and reported that the dysregulation of multiple groups of lipid metabolites was strongly associated with an increased risk of pelvic pain.^65^ In murine models, short-chain fatty acid levels are decreased in endometriosis.^66^^,^^67^ Several studies have compared the plasma metabolites in women with and without endometriosis, and have reported increased blood levels of lipids and amino acids and decreased levels of isoleucine and tryptophan in patients with endometriosis compared to the controls.^68^^,^^69^^,^^70^^,^^71^ These studies indicate that metabolic disturbances are closely associated with the occurrence and development of OvE. However, effective non-invasive biomarkers, including metabolites and clinical features that reflect the pathogenesis of OvE, have not been selected to construct a diagnostic model.

In summary, this study elucidated the serum metabolomic profile of OvE and characterized metabolites that were substantially altered in OvE. Subsequently, multiple machine learning algorithms were incorporated to facilitate the identification of meaningful metabolites, thereby enhancing the sensitivity and specificity of diagnostic indicators. A non-invasive diagnostic model with panel metabolites, CA125, and dysmenorrhea was developed that accurately distinguished between patients with OvE and HCs in a large validation cohort. These targeted metabolites could be used to predict OvE severity, particularly the degree of adhesion. Our findings highlight the pivotal role of metabolites in diagnosing and predicting OvE severity.

### Limitations of the study

There were certain limitations to our study. First, patients with OvE were classified as having an ASRM stage of III or IV in this study. That is because most of the OvE patients that we recruited in our hospital are admitted for surgery. Therefore, eligible patients are mostly in stages III and IV. It is challenging to diagnose patients with early-stage OvE using our metabolites diagnostic model. In future research, we will focus on the metabolomics of early-stage patients with OvE to refine this diagnostic model and facilitate the early detection of OvE. Second, the metabolite source is complex and is influenced by the host, gut microbiota, diets, and environmental pollution. Although we have controlled for the effects of hormones (in late proliferative) short-term diet (fasting over 8 h), and circadian rhythm (in early morning), the metabolic capabilities of the host also vary with these factors. Therefore, a more comprehensive view of the profiles of OvE would come from larger size of the cohorts. Third, our primary findings were based on an OvE cohort from the Women’s Hospital of Nanjing Medical University. Given that the serum metabolome is influenced by genetic, dietary, and environmental factors, multicenter investigations should be conducted in order to reduce population-specific bias. Fourth, we elucidated the diagnostic effects of the vital metabolites of OvE; however, the exploration of the functions of the selected metabolites in this study is only preliminary, and the detail functional and mechanistic aspects require further investigation. Despite these limitations, our results provide insights into the diagnostic and therapeutic potential of serum metabolites using untargeted and targeted metabolomics.

## Resource availability

### Lead contact

Requests for further information should be directed to and will be fulfilled by the lead contact, Dr. Pengfei Xu (pengfeixu@njmu.edu.cn).

### Materials availability

This study did not generate new unique reagents.

### Data and code availability

• The metabolomics data are available in the Metabolomics Workbench database under accession number ST003579 (https://doi.org/10.21228/M8353P).
• This paper does not report original code, but it is available from the lead contact upon request.
• Any additional information required to reanalyze the data reported in this work paper is available from the lead contact upon request.

## Acknowledgments

This project was supported by the 10.13039/501100001809National Natural Science Foundation of China (grant nos. 82472707 and 82201815), Nanjing Key Laboratory of Female Fertility Preservation and Restoration (no. 01002213), 10.13039/501100019349Nanjing Medical Science and Technique Development Foundation (grant no. YKK24153), and Research Innovation Program for Graduates of Jiangsu Province (SJCX24_0755). We would like to sincerely acknowledge Dr. Feifei Xu from the School of Pharmacy, Nanjing Medical University, for his valuable assistance in the mass spectrometry analysis.

## Author contributions

C.D., data curation, formal analysis, investigation, visualization, and writing – original draft; Y.C., formal analysis, software, methodology, writing – review and editing; Y.X., data curation, investigation, visualization, validation, and funding acquisition; M.L., software, and visualization; G.L., resources; S.X., resources and funding acquisition; N.Y., investigation; C.S., supervision; T.F., investigation; X.J., funding acquisition, project administration, supervision, and resources; P.X., conceptualization, resources, supervision, validation, project administration, and writing – review and editing.

## Declaration of interests

The authors declare no conflicts of interest.

## Declaration of generative AI and AI-assisted technologies in the writing process

During the preparation of this article, the authors used Deepseek to improve the readability of some texts. After using this tool, the authors reviewed and edited the content as needed and took full responsibility for the content of the publication.

## STAR★Methods

### Key resources table

**Table:** 

REAGENT or RESOURCE	SOURCE	IDENTIFIER
**Antibodies**		

Anti-Ki67	Novus Biologicals	Cat# NB500-170, RRID: AB_10001977
Anti-COX2	Cell Signaling Technology	Cat# 12282, RRID: AB_2571729
Anti-F4/80	Proteintech	Cat# 28463-1-AP, RRID: AB_2881149
Anti-VEGFA	Proteintech	Cat# 66828-1-Ig, RRID: AB_2882171

**Biological samples**		

Sprague–Dawley of female rats	GemPharmatech Co., Ltd	N/A
Blood samples	Women’s Hospital of Nanjing Medical University	Metabolomics Workbench (ST003579): https://doi.org/10.21228/M8353P

**Chemicals, peptides, and recombinant proteins**		

Estradiol benzoate	Sigma	Cat# E8875
Penicillin G sodium salt	Beyotime	Cat# ST2560
Gallic acid	Sigma-Aldrich	Cat# G7384
Hippuric acid	Sigma-Aldrich	Cat# 112003
Ascorbic acid	Sigma-Aldrich	Cat# A92902
(R)-3-Hydroxybutyric acid	Aladdin	Cat# R304188
4-Methylumbelliferyl sulfate potassium salt	Aladdin	Cat# M131130
Alanine	Macklin	Cat# L800640
Arginine	Macklin	Cat# L800637
Aspartic Acid	Macklin	Cat# L767520
Beta-Inosine	Aladdin	Cat# I104348
Ellagic acid	Macklin	Cat# E808704
Fraxetin	Macklin	Cat# F809828
Isofraxidin	Macklin	Cat# I811611
Lactic acid	Sigma-Aldrich	Cat# L6661
Magnolol	Macklin	Cat# M813634
Melatonin	Aladdin	Cat# M118674
Myosmine	Aladdin	Cat# M342525
Indole-3-acetyl-valine	Macklin	Cat# N981778
N-Acetyl-aspartic acid	Macklin	Cat# N873432
N-Acetyl-DL-tryptophan	Aladdin	Cat# A100466
Quinic acid	Sigma-Aldrich	Cat# 138622
Sebacic acid	Aladdin	Cat# S108452
Phosphate-buffered saline	Gibco	Cat# 10010023
TRIzol Reagent	Invitrogen	Cat# 15596018CN

**Critical commercial assays**		

E2 ELISAKit	Houston, US	Cat# E-EL-0152
Reverse transcription kit	Vazyme Biotech	Cat# R323-01
SYBR Green dye method	Vazyme Biotech	Cat# Q711-02
Immunohistochemical staining kit	Proteintech	Cat# PK10006

**Deposited data**		

Metabolomics data	This study	Metabolomics Workbench (ST003579): https://doi.org/10.21228/M8353P

**Software and algorithms**		

Q Exactive HF-XQuadrupole-OrbitrapMS System	ThermoFisher	https://www.thermofisher.cn/
Triple Quad 6500+Mass Spectrometer	AB SCIEX	https://sciex.com/products/mass-spectrometers/triple-quad-systems/triple-quad-6500-system
StepOne real-time quantitative PCR system	ThermoFisher	https://www.thermofisher.cn/
Image-Pro Plus 6.0 software	Media Cybernetics	https://mediacy.com/image-pro/
GraphPad Prism 9	GraphPad Software	https://www.graphpad.com/
SPSS Statistics 20	IBM	https://www.ibm.com/products/spss-statistics
R project	4.2.0	https://cran.r-project.org/
MetaboAnalystR	4.0.0	https://www.metaboanalyst.ca/docs/RTutorial.xhtml
dcurves	0.4.0	https://cran.r-project.org/package=dcurves
WGCNA	1.73	https://cran.r-project.org/web/packages/WGCNA/index.html
pROC	1.18.0	https://cran.r-project.org/package=pROC
umap	0.2.10.0	https://cran.r-project.org/web/packages/umap/index.html
OptimalCutpoints	1.1–5	https://cran.r-project.org/web/packages/OptimalCutpoints/index.html
circlize	0.4.16	https://cran.r-project.org/web/packages/circlize/index.html
glmnet	4.1_4	https://cran.r-project.org/package=glmnet
rms	6.3–0	https://cran.r-project.org/package=rms
survival	3.3–1	https://cran.r-project.org/package=survival
python	3.11	https://www.python.org/
numpy	1.23.5	https://pypi.org/project/numpy/
pandas	2.1.4	https://pypi.org/project/pandas/
sklearn	1.3.0	https://pypi.org/project/scikit-learn/
matplotlib	3.8.0	https://pypi.org/project/matplotlib/
scipy	1.11.4	https://pypi.org/project/scipy/
seaborn	0.12.2	https://pypi.org/project/seaborn/
shap	0.44.0	https://pypi.org/project/shap/
xgboost	1.7.3	https://pypi.org/project/xgboost/
tensorflow	2.12.0	https://pypi.org/project/tensorflow/
keras	2.12.0	https://pypi.org/project/keras/
scikit-learn	1.3.0	https://pypi.org/project/scikit-learn/

### Experimental model and study participant details

#### Clinical cohort

Initial discovery (*n* = 68, 34 HC vs. 34 patients with OvE) and subsequent validation (*n* = 237, 130 HCs vs. 107 patients with OvE) cohorts for untargeted and targeted metabolomic analyses were obtained from the Women’s Hospital of Nanjing Medical University. Patients diagnosed with OvE scheduled for surgery between December 25, 2021, and June 27, 2023, were recruited. All patients with OvE underwent laparoscopy, and the OvE diagnosis was confirmed by pathologists. During the same period, HC volunteers who underwent physical examinations after menstruation ended at our hospital were recruited. These females had no chronic illnesses, history of medications, or abnormalities on pelvic examination. The exclusion criteria were (1) pregnancy or lactation; (2) age <18 or >50 years old; (3) history of drug treatment within 3 months; (4) patients with a condition complicated by other diseases (e.g., hyperthyroidism, hypothyroidism, diabetes mellitus, or hypertension); (5) unwillingness to participate in the study or uncooperative with the researchers; and (6) unavailability of clinical data from the cohort. All participants are Chinese ethnicity and provided written informed consent, and all human studies were conducted following the Declaration of Helsinki. Blood samples were collected from the patients with OvE before surgery. All participants donated their blood samples in the early morning after fasting overnight for over 8 h. Samples were centrifuged immediately, and the upper serum was separated and stored at −80°C. The experimental protocols of this study were approved by the Ethics Committee of the Women’s Hospital of Nanjing Medical University. The methods were performed in accordance with the approved guidelines by the Ethics Committee of the Women’s Hospital of Nanjing Medical University (No. 2022KY-100).

#### Animals

The animals used in this study were all of female Sprague–Dawley (SD) rats (six-week-old), weighing 180–200 g. Rats were purchased from GemPharmatech Co., Ltd, Jiangsu (Jiangsu, China) and held under specific pathogen-free conditions. The animals were housed in a temperature- (22 ± 2°C) and humidity-controlled vivarium with lights maintained on a 12:12 light/dark cycle. All animal experiments were conducted in accordance with the institutional standard guidelines of Nanjing Medical University (approval number: IACUC-2212033).

### Method details

#### Untargeted metabolomic measurements

Untargeted metabolomic measurements of serum were performed using the Metabolon Discovery HD4 platform. Briefly, this platform is comprised of four independent ultra-high-performance liquid chromatography–tandem mass spectrometry (UPLC-MS/MS) methods: two separate reverse-phase (RP)/UPLC-MS/MS methods with positive ion mode electrospray ionization (ESI), RP/UPLC-MS/MS with negative ion mode ESI, and hydrophilic interaction liquid chromatography (HILIC)/UPLC-MS/MS with negative ion mode ESI. Metabolites with <10% missing values were included, and missing values were imputed using half the minimum within each metabolite.

#### Targeted metabolomic measurements

To determine the relative levels of selected metabolites, the same reference pool was used to normalize the abundance of each individual. The ExionLC AC system was connected to a 6500 QTrap Mass Spectrometer (Sciex) and operated in separate ion modes. The mobile phase and column used for RP liquid chromatography were the same as those used for untargeted metabolite profiling. The injection volume was 10 μL for each sample. The dwell time for each transition was 10 ms, a medium collision gas, the curtain gas was 40 psi, the ion spray voltages were 5,000 and −4,500 V, and the source temperature was 450°C. The metabolites were eluted from the column at a flow rate of 0.3 mL/min with gradually increasing concentrations of mobile phase B (initially 12% and 60% after 2.5 min). Linear 60–85% and 85–100% phase B gradients were set at 6 and 8.5 min, respectively. Quality control samples for targeted analysis were pooled: N-pool: C-pool (1:1). The declustering potentials and collision energies were optimized using the quality control samples from the control group. Metabolite peaks were integrated using Sciex Analyst 1.6.3 software. The selected metabolites standard sources and MS parameters for targeted metabolomics are as shown in Table S1.

#### OvE rat model experimental design

Rats were acclimated to the feeding regimen for one week prior to surgery. Then the rats were subcutaneously injected with estradiol benzoate (100 mg/kg) on day −7^20^^,^^21^.OvE was induced by autologous transplantation of left uterine endometrial tissue onto the surface of the right ovary according to a previous established protocol with little modifications,^72^ ensuring secure attachment. Following surgery, all rats were housed individually and received intramuscular penicillin injections for one week as infection prophylaxis. The rats were then randomly assigned to four groups, each containing five rats: the control, GA, AA, and HA groups. Three weeks post-surgery, rats in each group received intraperitoneal injections of the corresponding metabolites (GA: 100 mg/kg/day,^73^ AA: 200 mg/kg/day,^74^ HA: 100 mg/kg/day^31^) or an equal volume of phosphate-buffered saline (PBS). These metabolites were purchased from Sigma-Aldrich (St. Louis, MO, USA). The metabolites treatment lasted for six weeks, after which the rats were euthanized, and both ovaries were collected for analysis. All animal experiments were conducted in accordance with the institutional standard guidelines of Nanjing Medical University (IACUC-2212033).

#### Enzyme-linked immunosorbent assay (ELISA)

The serum concertation of OvE rats were detected using an E2 (Estradiol) ELISA Kit according to the manufacturer’s instructions (E-EL-0152, Houston, USA). Briefly, serum was obtained by centrifuging whole blood to separate the supernatant. We determined the number of microplate strips required for all samples (test, standard, and blank groups), each in triplicate. Firstly, 50 μL of standard working solution or test samples were added to each well. Subsequently, 50 μL of biotin-labeled antibody working solution was added to each well. The plate was sealed and incubated at 37°C for 45 min. The plate was then spun dry and washed four times with washing solution. Then, 100 μL of enzyme working solution was added to each well. The plate was sealed and incubated at 37°C for 30 min and then washed four times. Following this, 90 μL of chromogenic agent was added to each well under light-protected conditions. The plate was sealed and incubated at 37°C in the dark for 15 min. Finally, 50 μL of stop solution was added to each well, after which the OD value was immediately measured at 450 nm with an ELISA reader. A standard curve was generated using a four-parameter logistic model.

#### qPCR

Total RNA extraction was conducted using TRIzol reagent (according to the manufacturer’s instructions), and RNA concentration was measured using spectrophotometric absorbance, ensuring that the A_260_/A_280_ ratio was between 1.8 and 2.0. Reverse transcription of RNA to cDNA was carried out using a reverse transcription kit (R323-01, Vazyme Biotech Co., Ltd, Nanjing). QPCR was conducted using the Step One real-time quantitative PCR system, using the SYBR Green dye method (Q711-02, Vazyme Biotech Co., Ltd, Nanjing). Specific primers (Table S2) were used to amplify the target genes, with *β-Actin* serving as the housekeeping gene. Relative gene expression levels were calculated using the 2^−ΔΔCt^ method, and statistical analysis was conducted using inter-group comparisons.

#### IHC

Ovarian tissue sections were prepared using the conventional paraffin embedding method at a thickness of 4 μm. After deparaffinization and rehydration with ethanol gradients, antigen retrieval was performed using high-temperature treatment with antigen retrieval buffer, followed by natural cooling of the sections to room temperature. The sections were then incubated with 3% H_2_O_2_ at room temperature for 10 min to remove endogenous peroxidase activity. Afterward, the sections were blocked with 5% normal goat serum for 1 h to prevent nonspecific binding, and then incubated overnight with diluted primary antibodies. Horseradish peroxidase (HRP)-conjugated secondary antibody was subsequently added and incubated, followed by 3,3′-diaminobenzidine (DAB) staining and hematoxylin counterstaining. After dehydration using an ethanol gradient, the sections were mounted with neutral resin. Immunohistochemical staining for each sample was performed under identical conditions to ensure experimental consistency.

### Quantification and statistical analysis

Differences in baseline characteristics between patients and controls were analyzed using the *Chi-square* test or Fisher’s exact test for categorical variables and paired *t*-tests or Wilcoxon’s signed-rank test for continuous variables. Correlation coefficients between the metabolites were obtained using Spearman’s correlation analysis. To improve normality, the metabolite data were log-transformed. The dimensionality of the multivariate raw data was reduced using PCA to analyze the grouping, trends, and outliers of the observed variables in the dataset. The VIP values of the first two principal components of the OPLS-DA were combined with the results of FC and *t-*tests to screen differential metabolites. Metabolites with FC > 2, VIP >1.5, and *p* < 0.05 were further selected. MSEA and KEGG pathway analysis of the differentially expressed metabolites were conducted.

WGCNA is an effective tool for investigating highly coexpressed clusters of genes (modules), and its application has been extended to other types of -omics data. This method was used with a robust measure of correlation (bi-weight miscorrelation) to identify groups of correlated metabolites, called metabolite modules, which reflect a scale-free network topology of all measured metabolites. The association between each module and clinical features of OvE was evaluated using univariate logistic regression, with *p* < 0.05 considered statistically significant.

To examine the predictive performance of the metabolites, ROC analysis was calculated. Metabolites with AUC >0.7 and those in the black module were selected for further validation in the 107 patients with OvE and 130 HC individuals. All 237 samples were combined into the training and test sets at a ratio of 7:3 to avoid overfitting. Similarly, 21 metabolites plus 13 clinical markers were selected as the characteristics for developing a predictive model. Five machine-learning algorithms: CNN, NB, RF, SVM, and LASSO-logit were applied to assess the effectiveness and stability of these metabolites for diagnosis. Hyperparameter optimization was used in model development in our study. In detail, hyperparameter optimization based on random search were performed. During the random search process, 5-fold cross-validation was used to evaluate the model performance for each hyperparameter combination. ROC and PRC analyses were used to validate the diagnostic efficiency for OvE.

In experiments with two groups, a two-tailed paired *t* test was employed to analyze data from *in vivo* experiments. *p* < 0.05 was considered significant. All data are presented as mean ± SEM. For quantitative estimation of IHC, the staining results were semi-quantitatively scored by two independent observers and analyzed using Image-Pro Plus 6.0 software (Media Cybernetics, Silver Springs, MD, USA).
