# Supplementary material for: Serum metabolic fingerprinting for diagnosis and therapeutic applications of ovarian endometriosis
Source: iScience. 2026 Feb 2;29(3):114887. doi: 10.1016/j.isci.2026.114887 (PMC12930043; doi:10.1016/j.isci.2026.114887)
Supplement: Document S1. Figures S1–S9 and Tables S1 and S2 [file mmc1.pdf]

iScience, Volume *29*

Supplemental information

Serum metabolic fingerprinting for diagnosis and therapeutic applications of ovarian endometriosis

Chencheng Dai (戴晨诚), Yiwei Cao (曹怿玮), Yiran Xu (许逸然), Moyuan Li (李蓦圆), Guangquan Liu (刘光泉), Sujuan Xu (徐苏娟), Nuo Ye (叶诺), Changxiang Shi (施长香), Tiantian Fan (范湉湉), Pengfei Xu (许鹏飞), and Xuemei Jia (贾雪梅)

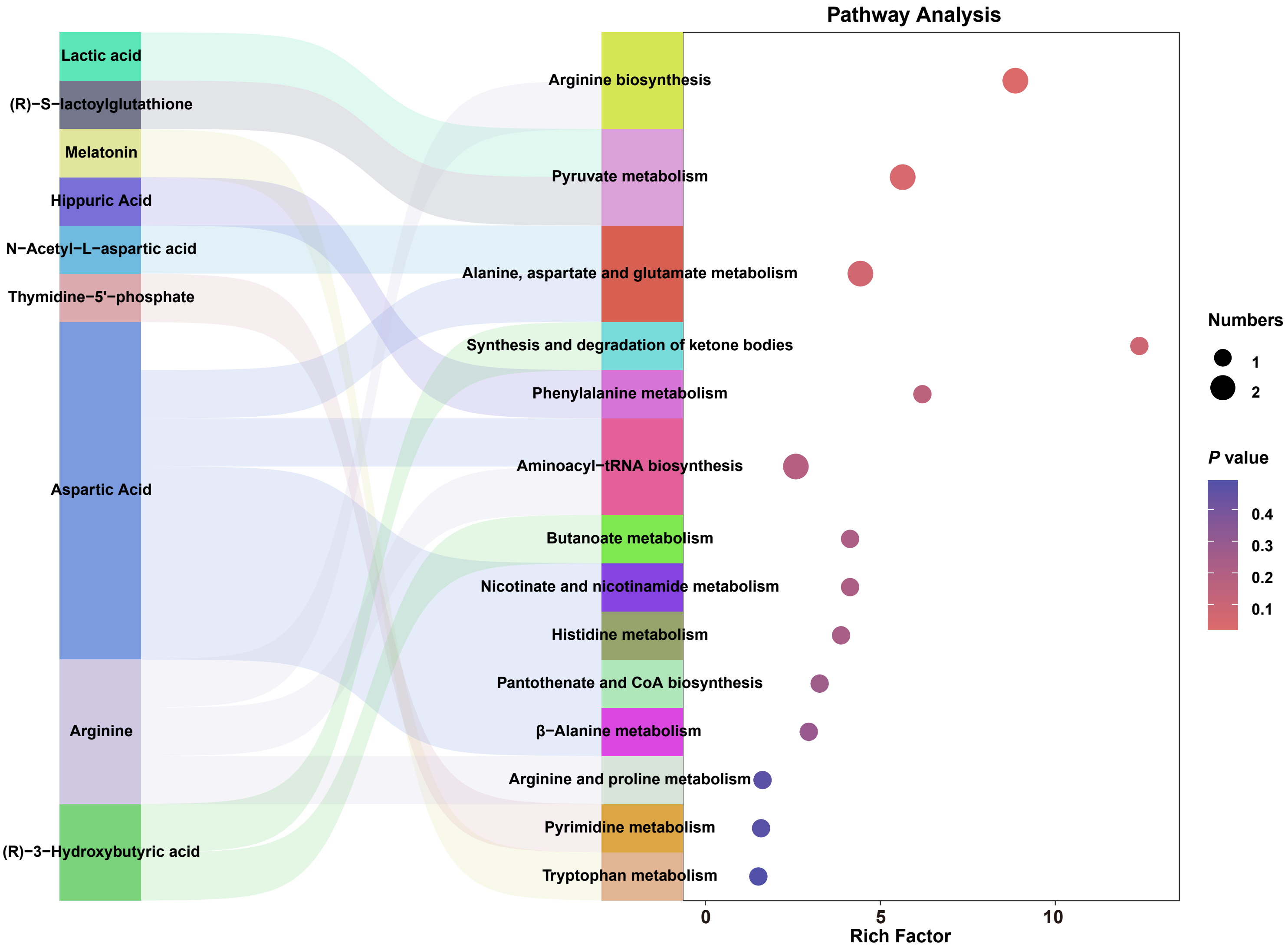

Figure S1. Sankey plot focused on the differential metabolites and pathways. Leftmost side represents metabolites, and the rightmost side represents related pathway of these metabolites.

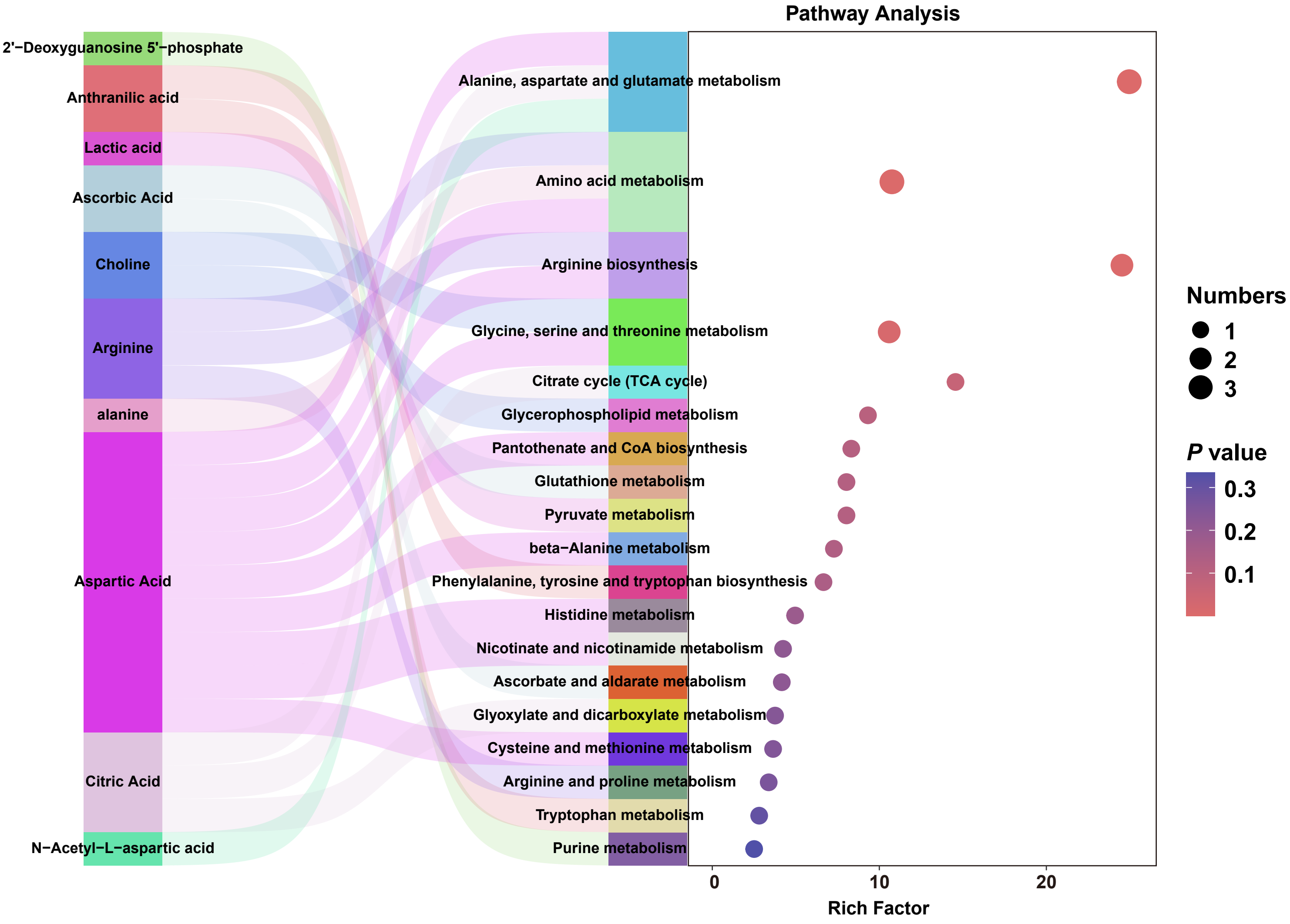

Figure S2. Sankey plot of the metabolites in black module and pathway. Leftmost side represents the metabolites in black module, and the rightmost side represents related pathways of these metabolites.

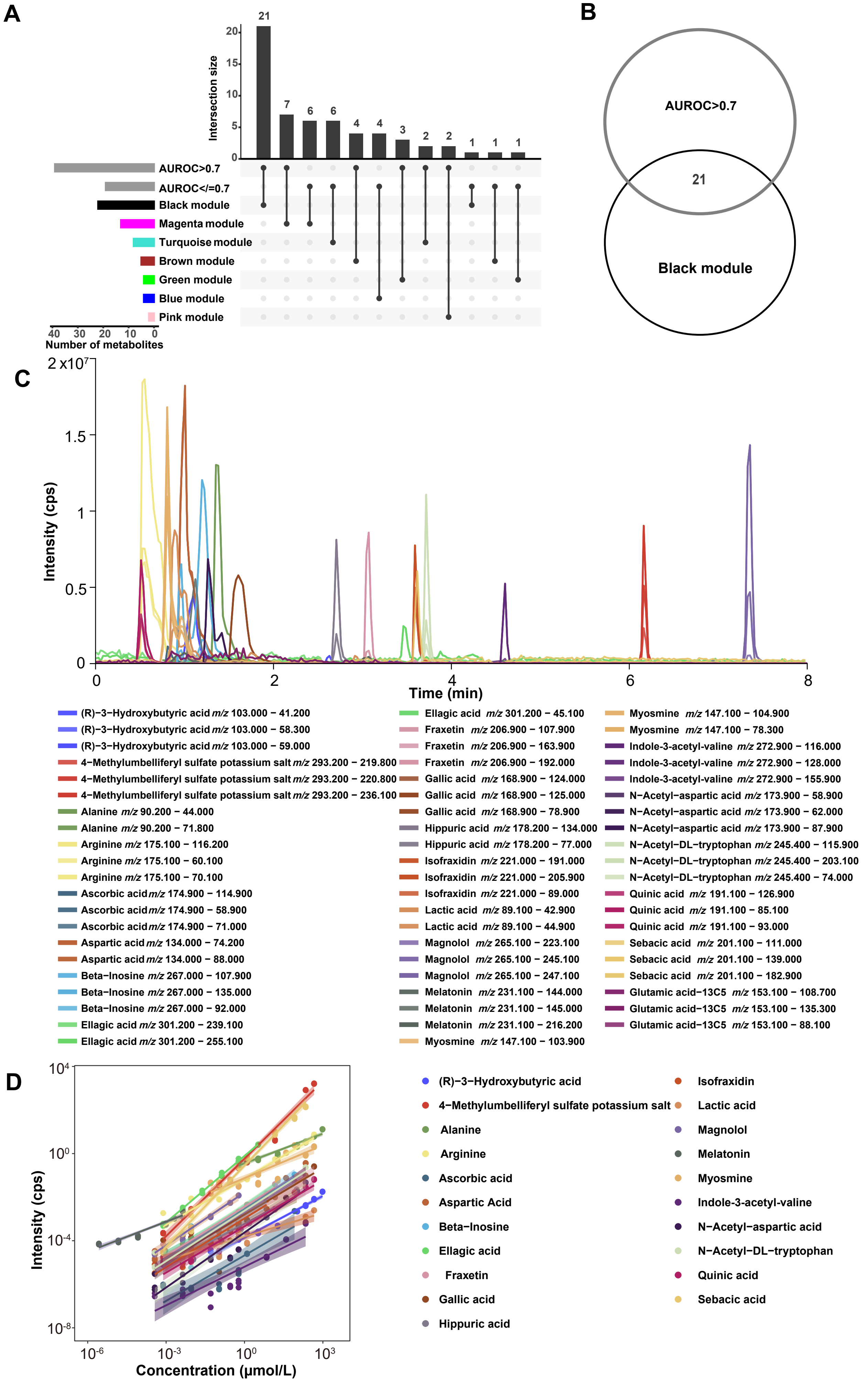

Figure S3. Selected 21 metabolites performed with targeted metabolomics. (A&B) The UpSet plot(A and Venn diagram(B) highlighting the 21 intersected metabolites between the metabolites in black module and AUROC > 0.7 in untargeted metabolomics. Supplementary refer for Figure 4A; (C) A typical MRM chromatogram of 21 target metabolites at serum samples; (D) Calibration Curve of 21 target metabolites.

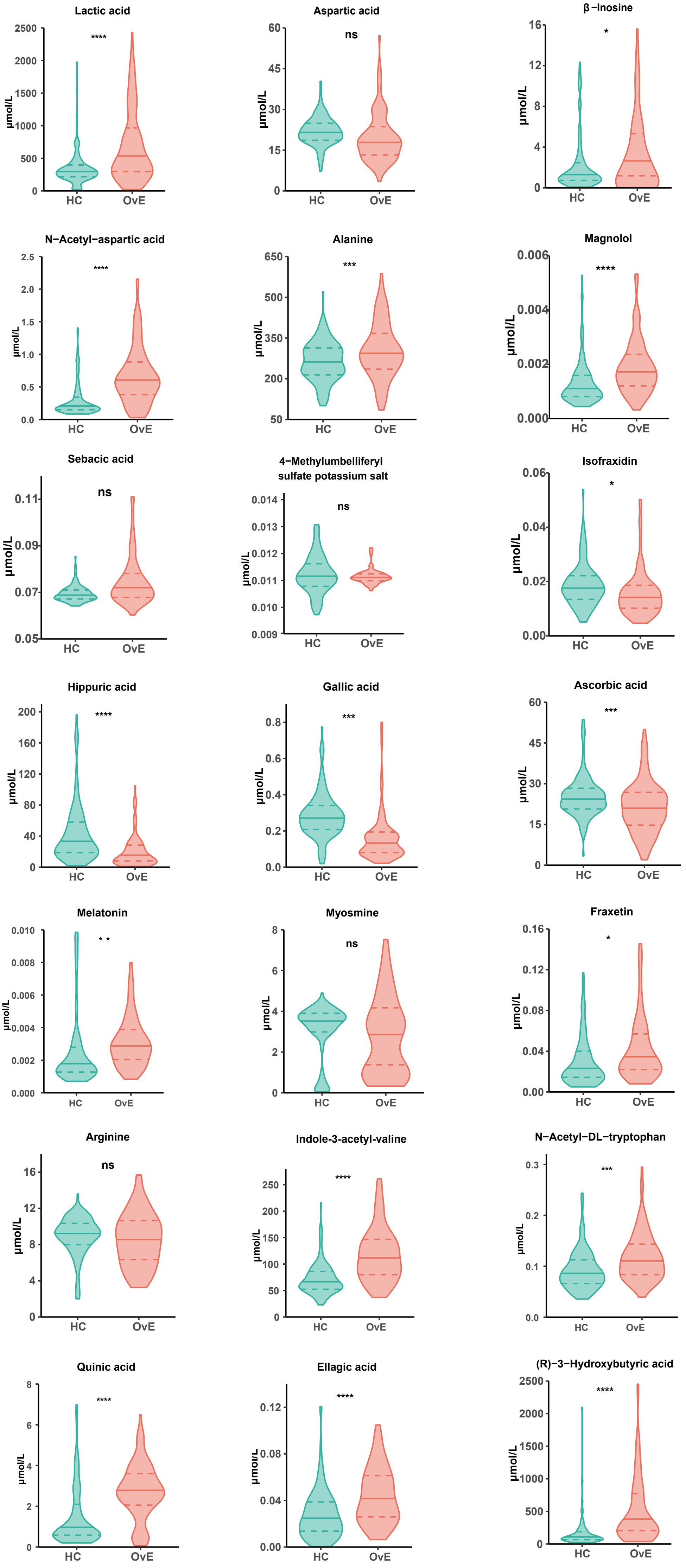
Lactic acid
****
Aspartic acid
ns
β−Inosine
*
N−Acetyl−aspartic acid
****
Alanine
***
Magnolol
****
Sebacic acid
ns
4−Methylumbelliferyl sulfate potassium salt
ns
Isofraxidin
*
Hippuric acid
****
Gallic acid
***
Ascorbic acid
***
Melatonin
* *
Myosmine
ns
Fraxetin
*
Arginine
ns
Indole-3-acetyl-valine
****
N−Acetyl−DL−tryptophan
***
Quinic acid
****
Ellagic acid
****
(R)−3−Hydroxybutyric acid
****
μmol/L
HC
OvE

Figure S4. The concentration of 21 target metabolites in the HC and OvE groups. Asterisks indicate significance between groups: ****p < 0.0001, ***p < 0.001, **p < 0.01, *p < 0.05 and ns indicated no significant.

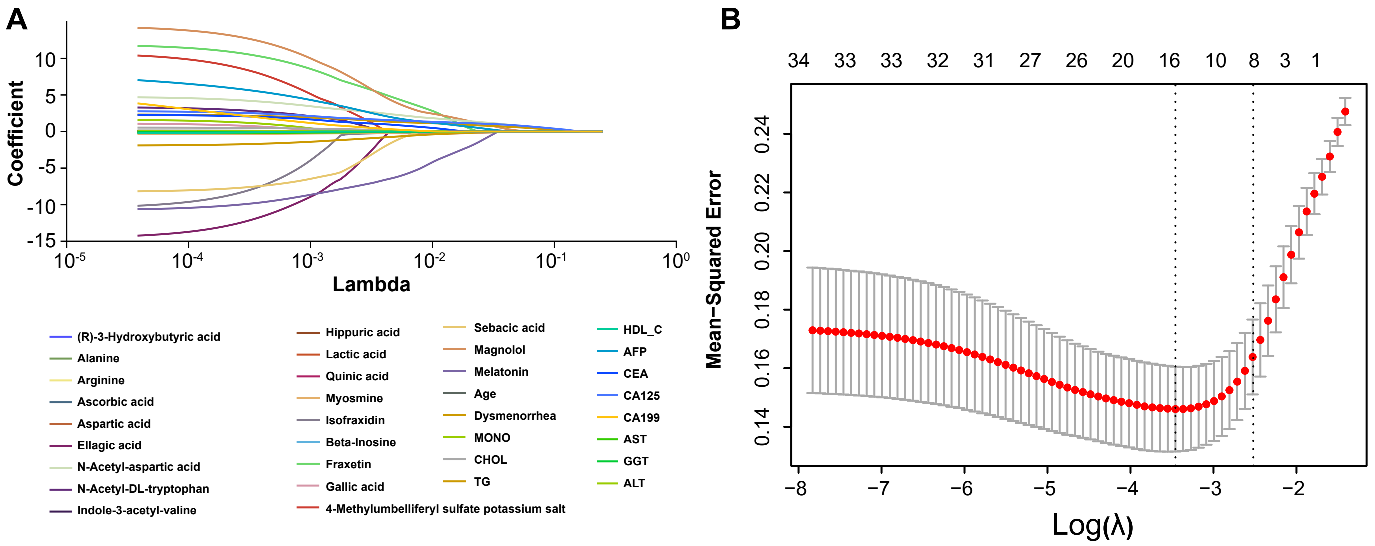

Figure S5. Metabolites selection by LASSO regression model. (A) LASSO regression coefficient path plot for metabolites and clinical indicators; (B) LASSO regression cross validation deviance. x-axis represents logλ values, y-axis represents deviance, red dots represent average deviance for each λ value, grey lines represent standard error of deviance, and vertical lines on x-axis represent optimal λ value.

A

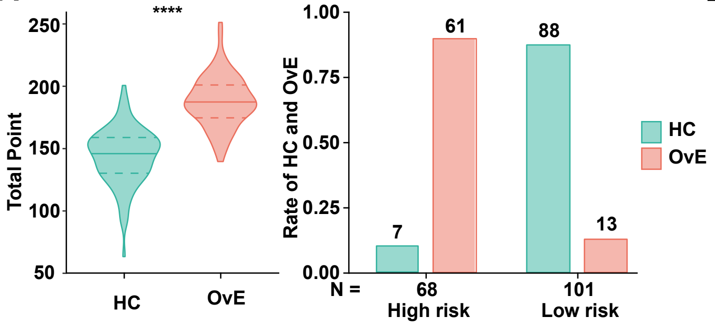

B

	High risk	Low risk
HC	7	88
OvE	61	13

C

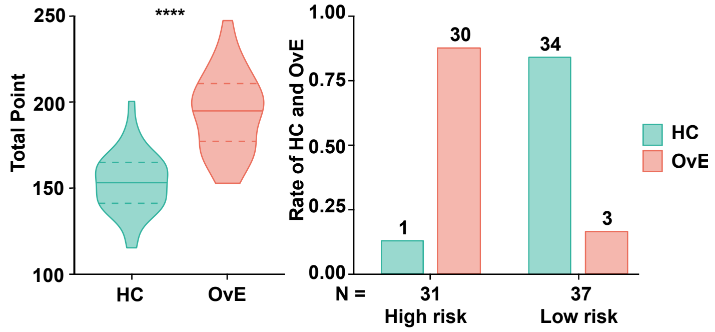

D

	High risk	Low risk
HC	1	34
OvE	30	3

Figure S6. The diagnostic model is validated by the confusion matrix diagram. Asterisks indicate significance between groups based on Wilcoxon test. **** $p < 0.0001$.

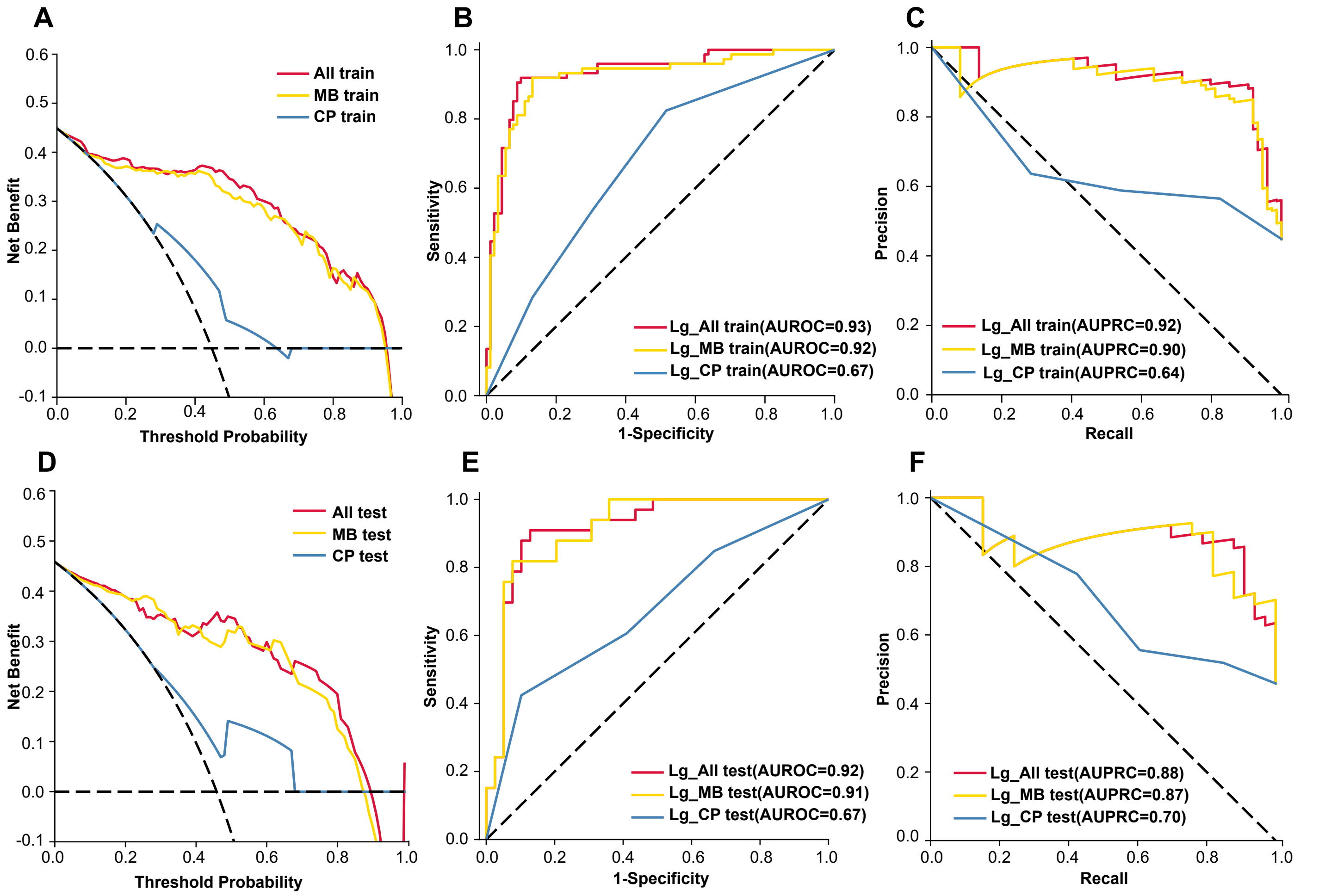

Figure S7. Diagnostic efficiency analyzed between six metabolites and two clinical features. (A) Decision curve analysis of six metabolites (MB), two clinicopathological features (CP) and combined metabolites and clinicopathological features (All) from training set; (B) The ROC curve of the MB(cutoff =0.428, sensitivity = 91.9%, specificity =86.8%), CP (cutoff =0.376, sensitivity =82.4%, specificity =48.4%)and All (cutoff =0.438, sensitivity = 91.9%, specificity =90.1%) from training set performance; (C) The PRC curve of the MB (cutoff = 0.428; recall = 91.9%, precision =85.0%), CP (cutoff = 0.376; recall = 82.4%, precision =56.5%) and All (cutoff = 0.438; recall = 91.9%, precision =88.3%) from training set performance; (D) Decision curve analysis of MB, CP and All from test set; (E) The ROC curve of the MB(cutoff =0.524, sensitivity = 81.8%, specificity =92.3%), CP (cutoff =0.579, sensitivity =42.4%, specificity =89.7%)and All (cutoff =0.458, sensitivity = 90.9%, specificity =87.2%) from test set performance; (F) The PRC curve of the MB (cutoff = 0.524; recall =81.8%, precision =90.0%), CP (cutoff = 0.579; recall = 42.4%, precision =77.8%) and All (cutoff = 0.458; recall = 90.9%, precision =85.7%) from test set performance.

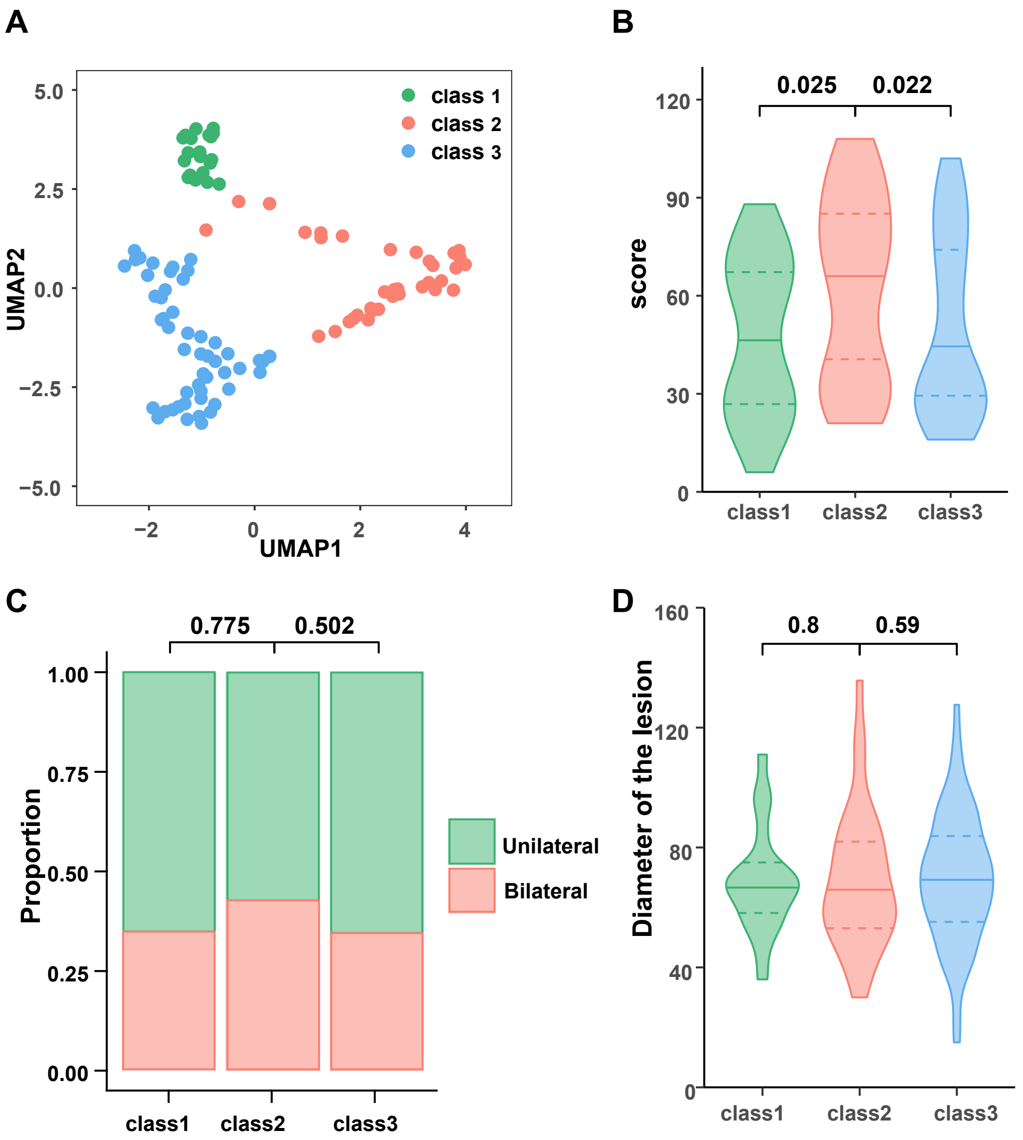

Figure S8. Prediction of the severity of OvE by selected metabolites. (A) OvE patients were divided into 3 classes based on the 21 target metabolites by UMAP; (B) The ASRM scores were performed by Wilcox among three classes; (C-D) The proportion of laterality (unilateral or bilateral) (C) and the diameter of the lesion (D) of ectopic endometrial lesions were analyzed by Fisher and Wilcoxon among 3 classes.

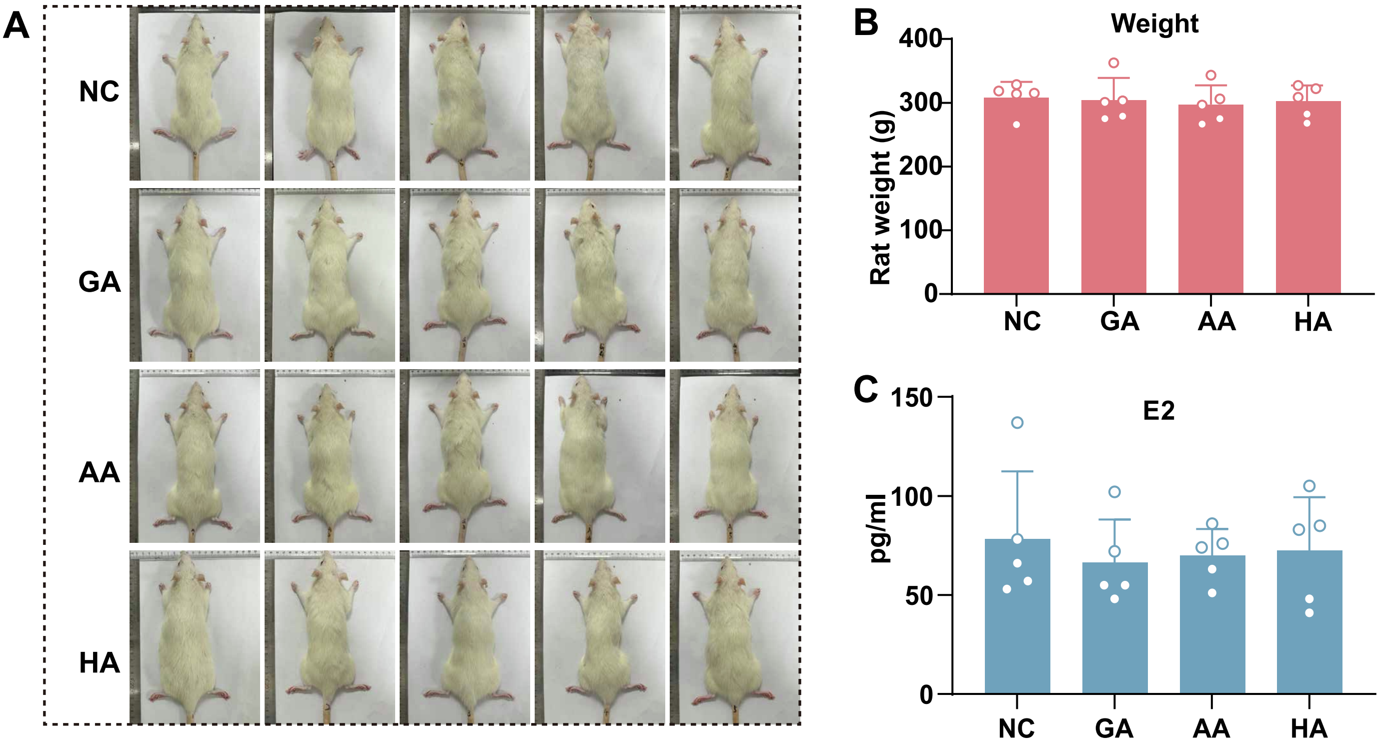

Figure S9. Selected metabolites treatment in rat OvE models. (A) The rats from NC, GA, AA and HA groups; (B) The weights of rats from NC, GA, AA and HA groups; (C) The serum estrogen concentration from NC, GA, AA and HA groups. There was not marked difference in body weight or serum E2 between the four groups based on the *t*-test.

Table S1. The selected 21 metabolites standard sources and MS parameters for targeted metabolomics

Metabolites	SOURCE	IDENTIFIER	Optimization of MS parameters						
			t_R /min	m/z (Q1)	DP / V	m/z ($Q3^1$)	CE^1/eV	m/z ($Q3^2$)	CE^2/eV
(R)-3-Hydroxybutyric acid	Aladdin	R304188	1.097	103.0	80	41.2	40.00	59.0	25.00
4-Methylumbelliferyl sulfate potassium salt	Aladdin	M131130	6.162	293.2	80	220.8	30.00	236.1	20.00
Alanine	Macklin	L800640	1.353	90.2	80	44.0	16.32	71.8	17.00
Arginine	Macklin	L800637	0.551	175.1	80	116.2	20.57	70.1	30.11
Ascorbic acid	Sigma	A92902	0.811	174.9	80	114.9	16.06	71.0	17.05
Aspartic Acid	Macklin	L767520	1.005	134.0	80	74.2	17.67	88.0	13.45
Beta-Inosine	Aladdin	I104348	1.197	267.0	80	107.9	52.47	135.0	29.42
Ellagic acid	Macklin	E808704	3.460	301.2	80	239.1	20.00	255.1	20.00
Fraxetin	Macklin	F809828	3.071	206.9	80	107.9	30.00	192.0	30.00
Gallic acid	Sigma	G7384	1.605	168.9	80	78.9	30.00	125.0	20.00
Hippuric acid	Sigma	112003	2.707	178.2	80	134.0	15.00	77.0	25.00

Isofraxidin	Macklin	I811611	3.590	221.0	80	191.0	30.00	205.9	20.00
Lactic acid	Sigma	L6661	0.889	89.1	80	42.9	15.00	44.9	15.00
Magnolol	Macklin	M813634	7.356	265.1	80	245.1	30.00	247.1	30.00
Melatonin	Aladdin	M118674	1.123	231.1	80	145.0	30.00	216.2	20.00
Myosmine	Aladdin	M342525	0.805	147.1	80	103.9	30.00	104.9	35.00
Indole-3-acetyl-valine	Macklin	N981778	4.603	272.9	80	116.0	22.91	155.9	20.84
N-Acetyl-aspartic acid	Macklin	N873432	1.263	173.9	80	58.9	20.00	87.9	20.00
N-Acetyl-DL-tryptophan	Aladdin	A100466	3.716	245.4	80	115.9	20.00	203.1	20.00
Quinic acid	Sigma	138622	0.511	191.1	80	85.1	30.00	93.0	30.00
Sebacic acid	Aladdin	S108452	3.616	201.1	80	139.0	21.79	182.9	201.10

t_R: Retention time; DP: Declustering potential; CE: Collision energy.

Table S2. The primer of rat used in this study

Gene	Forword (5'-3')	Reverse (5'-3')
β-actin	TCACCCACACTGTGCCCATCTATGA	CATCGGAACCGCTCATTGCCGATAG
Il-6	ACTTCACAAGTCGGAGGCTT	AGTGCATCATCGCTGTTCAT
Tnfa	GCGTGTTCATCCGTTCTCTAC	GTCTCGTGTGTTTCTGAGCAT
Nfkb1	GTGGGCAAGCACTGTGAGGA	TCATCCGTGCTTCCAGTGTTTC
Cyp19a1	ATGGGGATTGGAAGTGCCTG	TCATGAAGAAAGGGCGGACC
Ngf	CCAGCCTCCACCCACCTCTTC	GCTTGCTCCTGTGAGTCCTGTTG
Cox2	ATAGCAAATCCTTGCTGTTCCA	CTCCCTTGAAGTGGGTCAGG
Vegfa	GTGACAAGCCAAGGCGGTGAG	GATGGTGGTGTGGTGGTGACATG
Ccl5	ATATGGCTCGGACACCACTC	TGACAAAGACGACTGCAAGG
Il1b	TGACTTCACCATGGAACCCG	GACCTGACTTGGCAGAGGAC
